# Shaping the discourse on rehydrating children in heat stress: expositions from India

**DOI:** 10.3389/fped.2026.1895976

**Published:** 2026-09-18

**Authors:** Rajib Dasgupta, Shreya Pujari, Purnamita Dasgupta

**Affiliations:** 1 Centre of Social Medicine and Community Health, School of Social Sciences, Jawaharlal Nehru University, New Delhi, India; 2 Environmental and Resource Economics Unit, Institute of Ecohnomic Growth, Delhi, India

**Keywords:** heat action plans, heat related illnesses, oral rehydration salt (ORS), oral rehydration therapy, rehydratation

## Abstract

Heat health responses for young children constitute a unique public health challenge and Oral Rehydration Solution (ORS) have emerged as a key intervention in various advisories and heat action plans HAPs). ORS is an extraordinarily impactful first-line therapeutic intervention in reducing mortality and morbidity on account of dehydration and dyselectrolytemia in children caused by acute diarrheal diseases and is backed by state health and non-health agencies the world over. The reconstitution of ORS into a solution for oral rehydration therapy relies on the packaging instructions and frontline health workers; systematic reviews highlight low levels of knowledge and understanding of these aspects. Most of these HAPs and advisories are conspicuously silent on reconstitution and dosages. We have drawn upon available global literature and Indian evidence to shape this discourse across three strands – policy perspectives, clinical evidence and critiques on inappropriate usage of ORS and mislabeling of rehydration fluids to shape our arguments. It is imperative that only WHO formulated ORS is used for this purpose. Heat Action Plans need to specify age- and weight-specific ORS guidance, in line with the UNICEF advisory. India’s linguistic diversity calls for multilingual and pictorial instructions for correct preparation by caregivers, Capacity building of first responders and frontline health workers to correctly administer ORS for children’s HRIs is also an urgent imperative.

## Introduction

Heat stress is fast emerging as a leading cause of weather-related deaths; in specific, it may exacerbate associated comorbidities and increase the risk of accidents and transmission of some infectious diseases (1). The UNICEF estimates that 559 million children are exposed to high heatwave frequency globally while 624 million are exposed to one of three other high heat measures of duration, severity, or extreme temperature and by 2050 nearly every child may face more frequent heatwaves even if countries are on track to cap temperature increase as per the 2015 Paris Agreement (2).

India experienced the most intense heatwave year in 2024 and 71 per cent of respondents in a recent countrywide survey reported that they experienced severe heatwaves over the past year (3). Estimates suggest that 54 million students in India were affected by climate-related school disruptions in 2024 on account of climate hazards, principally on account of heatwaves (4). Heat waves are region-specific meteorological events based on departure from normal climatological values, with uncharacteristically high temperatures and local excess heat accumulating over days and nights (5, 6). Heat stress is marked by disruption of normal physiological functioning (disruption of thermoregulation) and heat related illnesses (HRIs) are specific clinical manifestations on account of heat stress (7). The evidence on infant mortality from heat in India is evolving; current estimates indicate that 2 children die as infants out of 1000 births and that 0–4 years is the most vulnerable age group for increased relative risk of all-cause mortality in association with extreme heat events (8).

ORS has a sterling legacy in the context of acute diarrheal diseases but the experience of its uses in heat stress, along with other hydration fluids, is emerging. Stocking ORS sachets in child care centers, schools and frontline health units is standard advisory now and health workers are required to promote its usage.

The adverse effects of inappropriate use of ORS, particularly overuse, are well known and the importance of aligning knowledge and practice in the context of acute diarrheal diseases especially in home settings has been extensively researched. This body of research puts the spotlight on barriers to uptake and ineffectiveness arising from factors such as delayed initiation, incorrect preparations, and caregiver compliance issues (9–11). Yet, there is a dearth of guidance, public interest communication, and supervision under which ORS is being administered to babies and children in managing HRIs or high heat exposure.

This perspective piece contributes to the discourse and provides insights regarding the appropriate use of ORS and the limitations and dangers of both inappropriate use of ORS and rehydration fluids that do not adhere to WHO specifications in heat-health contexts.

We have drawn upon relevant global literature and some specific Indian evidence to shape this discourse. We rely on information and data from policy discourse, clinical evidence and critiques on inappropriate usage of ORS to shape our arguments. We initiate the process by developing a comprehensive understanding of literature including review articles on this subject and develop an initial level of synthesis. We accessed standard databases including PubMed, Scopus, Web of Science and ScienceDirect for this purpose (12). We located several “seed” or seminal papers in this field and followed up with backward searching and forward searching strategies to shape our arguments (13). A key limitation was the dearth of specific materials on use of ORS in the heat-health context as this is an emerging field.

## The burgeoning market for ORS and rehydration drinks

ORS is an extraordinarily impactful first-line therapeutic intervention in reducing mortality and morbidity on account of dehydration and dyselectrolytemia in children caused by acute diarrheal diseases and is backed by state health and non-health agencies the world over (14, 15).

In the matter of heat related illnesses (HRIs) it is increasingly considered a key intervention to prevent and/or correct dehydration, particularly in children. At the same time a number of rehydration fluids, that are not WHO-ORS formulations, are available for over-the-counter (OTC) sale. ORS and rehydration drinks are fast emerging as a phenomenal commercial entity. The global market is expected to nearly double in the coming decade, from 5.87 USD billion in 2024 to 9.56 USD billion by 2035 (16), the leaders being FDC Limited, Cipla, Wallace Pharmaceuticals and Zydus Cadilla Healthcare Ltd (16). The moving annual turnover of ORS more than doubled in the last four years in India, up from Indian rupees (INR) 3,340 million in May 2020 to INR 7,160 million in May 2024 (17). Electral (FDC Limited) sales recorded 26%+ year-on-year increase in 2024 and was the 4th most sold medicine in April 2024, up from its 20th position in the previous month, signifying a substantial jump in sale of ORS and rehydration products as summer ensues (18).

ORS and rehydration products are therefore experiencing an intense phase of commercialization, the ill effects being wider over-the-counter use, increased costs with over and inappropriate use, unregulated marketing campaigns, marginalization of local health traditions and traditional preventive practices and lax regulatory oversight. In October 2025, the Food Safety and Standards Authority of India (FSSAI) took a step forward in prohibiting the naming of a food product ‘ORS’ unless it is based on the formula recommended by WHO.

## Physiological heat vulnerabilities for children

Infants and young children have specific heat-related physiological and behavioral vulnerabilities: (i) children produce more heat per kilogram of body weight; (ii) younger children have a higher surface area-to-mass ratio and absorb more heat from the environment; (iii) children have lower sweat rate per gland and begin to sweat only at a higher body temperature; (iv) infants experiencing heat stress have less capacity for regulating their temperature independently; (v) infants and young children have poorer blood circulation and lower proportional cardiac output and therefore are unable to adapt quickly to changes in temperature; and (vi) children tend to spend a lot of time outdoors and inadequately replenish fluid loss sustained during prolonged exposure and playing, leading to exertional heat illnesses. There are clear associations between pediatric emergency room visits and extreme heat events, mostly on account of dehydration, electrolyte disorders, gastrointestinal infections, and exacerbations of bronchial asthma (19).

Dehydration in the context of HRIs is generally assessed by urine color or volume but a robust point-of-care test to detect/measure dyselectrolytemia is not available. Consumption of plain water quenches thirst and provides short-term relief during dehydration but excessive heat leads to further water loss. ORS, an iso-osmolar glucose-electrolyte solution with base and citrate, helps replenish fluids and electrolytes lost due to sweating by helping the body absorb fluids more efficiently, thereby balancing the electrolyte levels and restoring homeostasis.

## Aligning rehydration to physiological needs

It is a standard practice of any pediatric and adolescent advisory to include specifications for different age and/or body weight groups. The UNICEF advisory specifies rehydration guidelines (20) for infants, older infants, and children, with instructions on age specific use of ORS (Box 1).

BOX 1 **Rehydration advisory for children.**

- “Infants under six months: Breastfeed to rehydrate the infant; encourage the mother to also drink more water, especially if breastfeeding.”
- Older infants and children: Water in small amounts to help them become used to it.
- If the child has sweated a lot or is sweating a lot, add some Oral Rehydration Salt (ORS) to the water for the child. Carefully follow the directions on the packet. If there are no directions, use the following guidance:
  - A child under the age of two years needs at least 1/4 to 1/2 of a large (250 mL) cup of the ORS drink
  - A child aged two years or older needs at least 1/2 to 1 whole large (250 mL) cup of the ORS drink
- If no premade ORS is available dissolve six level teaspoons of sugar and 1/2 level teaspoon of salt in 1 liter of clean water.

Older children: Add ORS to water if excessively sweating. They should drink 100 mL of ORS every 5 minutes until they seem better

Source: (20)

These advisories have not received much attention in general and India’s national and state guidelines on ORS use for HRIs management are also silent on this aspect.

National advisories and Heat Action Plans (HAPs) highlight the centrality of ORS in maintaining hydration during the summer season and particularly heat waves (20–22). Awareness materials (posters and videos) from India's National Disaster Management Authority (NDMA) includes recommendation on ORS along with other home-based hydrating fluids and making ORS available at health centers and Anganwadi Centers (child care centers) (23). The National Program on Climate Change and Human Health (NPCCHH) (24) includes ORS as a key hydration tool for institutional management of HRIs and recommends distribution of ORS by the frontline health staff during hot days. In specific, these public advisory documents for HRIs suggest “oral rehydration with salt-containing fluid” for the pediatric age groups – specifically mentioning ORS and several home-based fluids: “ORS/ *lassi*/ *nimbu pani*/ lime water with salt and sugar/ rice water/ *dal* water/ coconut water/ etc.” (25).

There are critical learnings from widespread ORS use in acute diarrheal disease contexts, particularly in young children. The original WHO-ORS formulation had an osmolarity of 311mOsm/L and was associated with hypernatremia (with symptoms of seizures, excessive irritability and even death) among infants; subsequent revisions reduced osmolarity to 245 mOsm/L. Over-dilution of ORS has been linked to hyponatremia and hypokalemia (26, 27). Heat health related documents widely recommend ORS as a hydration measure but do not specify the osmolarity aspects and adverse effects of inappropriate use.

Commercially available ORS is generally based on WHO recommendations and needs to be dissolved in a specified amount of clean water. Most ORS brands specify osmolarity (245mOsmol/L) but some ‘rehydration’ or ‘electrolyte’ drinks such as, ORSL Electrolyte Drink (JNTL Consumer Health (India) Pvt. Ltd) or Enerzal (FDC) do not. The ready-to-use packages do not come with the need to dissolve them in water; the powder form of ORS does, but user guidance regarding the amount of water remains variable. Pediatricians are increasingly worried about sugar-rich beverages being marketed as ‘electrolyte drinks’ and potentially contributing to complication and deaths among children with diarrhea (28).

As an OTC formulation, the reconstitution of ORS into a solution for oral rehydration therapy (ORT) relies solely on the packaging instructions and the advisories by frontline health workers. Different brands have somewhat different directions for reconstitution though. Most packaging instructions in India are primarily in English with some exceptions such as that by Wallace Pharmaceuticals in Hindi. A sachet of Electral (FDC Limited) needs to be dissolved in “one liter of drinking water” while pediatric-specific ORS (Wallace Pharmaceuticals) requires “200 mL of previously boiled and cooled water”. In case, less water is used for dissolution, the solution will become over concentrated which will overload sodium in the body whereas use of more water than required will lead to solution becoming too dilute and thus, failing to replace lost electrolytes sufficiently. There is global evidence of both inter-packet variation as well as inter-caregiver variation leading to deviations in the resulting osmolarity of the ORS solution (29–31). When pre-packaged salts are dissolved in less than recommended water, the resulting higher salt concentration can lead to higher fractional excretion of sodium (FENa%>2%), indicating an excess sodium load being excreted by the kidneys on account of salt overload (hypernatremia).

Systematic reviews (in the acute diarrheal diseases context) point to low knowledge and understanding of ORT being widely prevalent in low-and-middle-income countries, and in specific low knowledge on ORT preparation (32). Case studies point to complications and deaths in young babies on account of caregivers dissolving the standard formulation in inappropriate quantities of water owing to variations in the size of ORS pouches and prescribers advising standard reconstitution directions (29). Inconsistent home-based preparation and powder reconstitution can result in hyperosmolar or hypo-osmolar solutions with inaccurate electrolyte or glucose concentrations (29). Reconstituting ORS to 1-liter solutions pose additional challenges in terms of dilution and dosing errors (and consequent efficacy) as well as potability (33).

An analysis of “liking of sweetness, saltiness, and thirst-quenching ability” during heat stress suggests an overall hedonic shift to saltiness, where ORS can play an appropriate role. There is robust evidence that heat wave conditions significantly disrupt child feeding practices and is a barrier in achieving minimum dietary diversity (MDD) and disrupting minimum meal frequency (MFF); infants under thermal stress, therefore, prioritize hydration needs over food and ORS can play a critical role in these windows (34). It is also critical to note that carbohydrates in non-ORS beverages can adversely impact fluid replacement (35). The core temperature of children rises faster than adults during rehydration and hence constitutes a different kind of urgency; it is important to replace the lost salt and hence the relevance of appropriate use of ORS or a homemade formula of 1 tsp salt in 500 mL of water (36). While most literature address concerns in home or play settings, mass gatherings and similar public events constitute additional risk contexts where paramedic or health worker led appropriate oral rehydration measures play a significant role (37).

## Efficient and equitable outcome: communication matters

ORT campaigns for acute diarrheal diseases have traditionally relied on a mix of communication campaigns and social marketing strategies (38). The focus was on behavior change with less attention paid to skill training (39). There are models of success when simple messages are complemented with demonstration of steps of preparing and administering ORT (40). Another key learning from rural LMIC contexts is that while the instructions for reconstitution are in English and the instructions are read or explained to the purchaser, it is not mere “awareness of its benefits” but an understanding of the environmental and biological causes of the underlying condition that enables awareness to translate to actual use and treatment (41). Low levels of knowledge and understanding in preparing ORT is a well-known barrier but a less recognized one is the perceived “ambivalent status of ORS as a non-exclusive, low-technology, inexpensive medicine” (32). A key programmatic barrier therefore, beyond access issues, is limited training and re-training of frontline health workers and their limited communication skills (42). Contemporary dimensions of health literacy in the context of ORS include making instructions for reconstitution that cater to varied literacy levels, language, culture, and cognitive and perceptual abilities; co-creation and co-evaluation of educational materials with target audience members; and, more imaginative use of pictographs (43).

Information, Education and Communication (IEC) materials of national programs and national/state Heat Action Plans (HAP) currently provide insufficient guidance regarding these critical guideposts. A recent national evaluation of the IEC campaigns undertaken by the Ministry of Health and Family Welfare, India noted significant challenges: (i) IEC materials are often based on reactive approaches and there is a lack of formative studies that can generate evidence to inform the IEC strategy/plan; (ii) access to radio is limited in current contexts; (iii) access to smartphones is variable across different regions of the country; (iv) varied viewership and preference for watching TV channels; (v) little or no operational specifications about where to display and how to use a particular IEC material for frontline teams; (vi) community needs assessment or media plan at a local level was more an exception than a rule; (vii) IEC materials received and pasted in health facilities hardly evidenced any interest or behavior change; and (vii) the role of IEC officers was limited to routine acts of sending and receiving IEC materials (44).

## In conclusion

The WHO strongly advocates community engagement as “a process of developing relationships that enable stakeholders to work together to address health-related issues and promote well-being to achieve positive health impact and outcomes,” (45) though it remains a weak link in several high priority national health initiatives (46). The community's role remains limited as passive participants. Active engagement is necessary in a citizen-science framework for co-creating (collaborative approach of creative problem solving between diverse stakeholders at all stages of an initiative, from the problem identification and solution generation to implementation and evaluation), co-designing (active collaboration between stakeholders in the design of solutions to a pre-specified problem) and co-producing (implementing previously determined solutions to a previously agreed problem with emphasis on the most efficient use of existing resources and assets) solutions (47).

We have sought to align the discourse within the contours of the Sustainable Development Goals (SDGs), in specific SDG 3 (Good Health and Well-being) and SDG 13 (Climate Action) in the spirit of responding to changing climate threats and institutionalizing appropriate responses. Heat health responses constitute public health and economic challenges and require planned responses that combine resilience building as well as emergency preparedness (48).

While ORS is a necessary intervention in management of HRIs, *ad hoc* and insufficient guidance may end up as avoidable complications for some babies that could be construed as public health iatrogenesis (49). The available body of evidence and norms of practice of ORS draws upon the clinical spectrum of acute diarrheal diseases that is contingent upon visible loss of fluids. In contrast, heat exhaustion and heat stroke in children set in more insidiously but precipitously; once a child has heat exhaustion, s/he can develop heatstroke in 30 minutes unless rapidly cooled down (50, 51). Guidance and community engagement for appropriate pediatrics use of ORS for heat stress is therefore both a technical and a moral imperative and calls for urgent attention.

We propose the following actionable interventions to distinguish WHO-formulated ORS from commercially available electrolyte beverages: (i) age- and weight-specific ORS guidance, in line with the UNICEF advisory, to be included within heat action plans; multilingual and pictorial preparation instructions, particularly given India’s linguistic diversity; (iii) conducting focused health literacy campaigns for public awareness; (iv) capacity building of first responders including frontline health workers such as ASHAs (community health workers) or Anganwadi Workers (Integrated Child Development Services functionaries), school teachers as well those in primary care clinical settings; and (v) appropriate regulatory and market interventions by relevant federal and state authorities.

## Data Availability

The original contributions presented in the study are included in the article/Supplementary Material, further inquiries can be directed to the corresponding author.
